# Comparative efficacy of topical povidone‐iodine and chlorhexidine gel on dental plaque regrowth in toddlers: A randomized controlled trial

**DOI:** 10.1002/cre2.755

**Published:** 2023-06-21

**Authors:** Nour Al Basha, Mawia Karkoutly, Nada Bshara

**Affiliations:** ^1^ Department of Pediatric Dentistry Damascus University Damascus Syrian Arab Republic

**Keywords:** antimicrobial, chlorhexidine, dental plaque, povidone iodine

## Abstract

**Objectives:**

This study aimed to compare and evaluate the efficacy of topical use of povidone‐iodine (PVP‐I) solution and chlorhexidine (CHX) gel on dental plaque regrowth after 3 and 7 days in toddlers aged 24–36 months.

**Materials and Methods:**

A randomized controlled trial that included 45 healthy toddlers aged 24–36 months, who were randomly assigned to three groups. The first group received a placebo (distilled water (DW)) (negative control). The second group received topical CHX gel (0.2% w/v) (positive control). The third group received topical PVP‐I solution (10% w/v). Plaque accumulation was measured at the baseline (t_0_), after 3 days (t_1_) and after 7 days (t_2_) using the Turesky‐modified Quigley–Hein plaque index (TMQHPI). Oral hygiene practices were prohibited during the trial period. The trial ID is ACTRN12623000567628.

**Results:**

In the DW group, the mean of the TMQHPI score was 1.89 ± 0.67 at t_0_ and decreased to 1.45 ± 0.66 at t_1_ (*p* = .028). Similarly, in the CHX group, the mean of the TMQHPI score was 1.83 ± 1.06 at t_0_ and decreased to 1.02 ± 0.99 at t_1_ (*p* = .033). Regarding the PVP‐I group, the mean of the TMQHPI score went from 1.84 ± 0.85 to 1.01 ± 0.61 at t_1_ and then increased to 1.57 ± 0.74 at t_2_. Those changes were statistically significant (*p* = .001) and (*p* = .002), respectively. No statistically significant difference was noted between TMQHPI scores at t_0_ (*p* = .789). Regarding t_1_ and t_2_, no statistically significant difference was found between the three groups (*p* > .05).

**Conclusion:**

CHX and PVP‐I efficacy lasted only for 3 days, and PVP‐I was not superior to CHX in terms of plaque control in toddlers. However, further studies are needed to determine the long‐term efficacy of these antiplaque agents in toddlers.

## 1 INTRODUCTION

Dental plaque is a microbial community that consists of microorganisms and their extracellular matrix sticking to dental surfaces and can be either supragingival or subgingival. Dental plaque accumulation is the initiating etiological factor for gingivitis and dental caries (Valm, 2019). There is a correlation between dental plaque accumulation and early childhood caries (ECC) in children aged 12–36 months. ECC is an alarming oral health issue and the most widespread chronic condition for both toddlers and infants (Meyer & Enax, 2018). However, it can be prevented by different prophylactic measures at various prevention levels including primary, secondary, and tertiary. Primary prevention measures aim to intervene at the youngest age possible before dental caries formation by caregivers, dental professionals, and the community (Meyer & Enax, 2018; Sitthisettapong et al., 2021). Antiplaque agents adjacent to mechanical plaque control are used as primary prevention methods. Hence, chemical plaque control agents should not be regarded as an alternative to mechanical methods. However, mechanical plaque control seems challenging for toddlers due to the lack of compliance and commitment on the part of parents (Jafer et al., 2016; Vyas et al., 2021).

Antiplaque agents suppress the growth of specific micro‐organisms and can be either bactericidal or bacteriostatic depending on their concentration (Vyas et al., 2021). Chlorhexidine (CHX), to date, is the gold standard in terms of chemical plaque control due to its antimicrobial efficacy and cationic structure. Its cationic property results in a phenomenon named substantivity, which means that CHX antimicrobial efficacy extended over a long period (Balagopal & Arjunkumar, 2013). In addition, CHX is biocompatible and safe for topical use in toddlers older than 2 months of age (Chapman et al., 2012). CHX is available in various forms, the gel type is easier to handle, provides a prolonged release, and has an analgesic effect (Ahmedi et al., 2023). Nevertheless, CHX has several adverse effects, including xerostomia, glossodynia, hypogeusia, tooth discoloration, epithelial desquamation, and parotid gland swelling (McCoy et al., 2008). Therefore, several antiplaque agents have been suggested to overcome the aforementioned drawbacks. Povidone‐iodine (PVP‐I) is a broad‐spectrum microbicidal agent that is economical and widespread (Eggers, 2019; Lepelletier et al., 2020). Furthermore, PVP‐I suppresses the levels of Streptococcus mutans and caries progression in young children (Amin et al., 2004). In addition, it is proven to be safe for topical use in toddlers older than 2 months of age (Chorney et al., 2020). However, it is contraindicated for patients with thyroid disorders or iodine allergies (Chundamala & Wright, 2007).

To the best of the authors' knowledge, no study has ever evaluated the efficacy of CHX gel and PVP‐I in terms of plaque control in toddlers. Hence, the aim of the current study was to compare and evaluate the efficacy of topical use of PVP‐I solution and CHX gel on dental plaque regrowth after 3 and 7 days, in toddlers aged 24–36 months.

## 2 MATERIALS AND METHODS

### 2.1 Study design and ethics

This was a three‐arm, double‐blind, randomized parallel‐group controlled trial. It was conducted from December 2022 to March 2023 at Damascus University. This study was approved by the ethics board at Damascus University (N 725/2022) and was conducted in accordance with Helsinki Declaration 2013 and CONSORT statement. Written informed consent was provided by the patients' legal guardians. This study was registered in Australian New Zealand Clinical Trials Registry (ACTRN12623000567628).

### 2.2 Eligibility criteria and recruitment

Inclusion criteria

1. Children aged 24–36 months.
2. Healthy children in terms of medical and dental status.
3. Children with full primary dentition.
4. Both male and female toddlers.

Exclusion criteria

1. Children with special health care needs (SHCN).
2. Children receiving any antibiotic therapy over the past 4 weeks.
3. Children with thyroid disorders or iodine allergies.
4. Children with CHX allergies.

**Figure 1:**
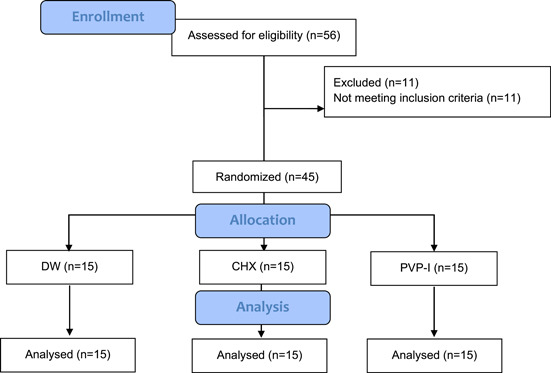
CONSORT flow diagram.

The CONSORT flow diagram is illustrated in Figure 1. Fifty‐six patients were assessed for eligibility. Based on selection criteria, 45 patients were assigned to the current study and were randomly allocated into three groups according to the antiplaque agent used:

Group 1: negative control or placebo, distilled water (DW) (*n* = 15).

Group 1: positive control, CHX gel (0.2% w/v) (PerioKIN, KIN Dental) (*n* = 15).

Group 2: PVP‐I solution (10% w/v) (Betadine Antiseptic, BETADINE®) (*n* = 15).

### 2.3 Sample size calculation

The sample size was determined using the G. Power 3.1.9 software (Heinrich‐Hein‐Universität‐Düsseldorf; http://www.gpow-er.hhu.de/). Effect size f = 0.4797282/α err prob = 0.05/Power (1‐β err prob) = 0.80/Number of groups = 3.

### 2.4 Randomization

Patients were randomized using randomization online software; http://www.randomization.com.

### 2.5 Blinding

This study was double‐blind since both the examiner and the statistician were blinded to group allocations. The examiner was unaware of which experimental arms participants have been assigned to and the statistician was kept unaware of which records belong to which interventional arm.

### 2.6 Clinical procedures and evaluation

The toddlers were randomly assigned to three groups. The first group received a placebo (DW) (negative control). The second group received topical CHX gel (0.2% w/v) (positive control). The third group received topical PVP‐I solution (10% w/v). Each antiplaque agent was applied using a cotton‐tipped swab on tooth surfaces in the clockwise direction. It was applied for one time due to the lack of compliance on the part of toddlers. Plaque accumulation was measured at the baseline (t_0_), after 3 days (t_1_) and after 7 days (t_2_) using the Turesky‐modified Quigley–Hein plaque index (TMQHPI). Dental plaque was disclosed using the two‐tone erythrosine‐free plaque disclosing dye solution Mira‐2‐Ton® (Hager & Werken). The toddlers' legal guardians were instructed to avoid feeding their child for 30–60 min postapplication and oral hygiene practices were prohibited during the trial period. TMQHPI scores are as follows (Quigley & Hein, 1962; Turesky et al., 1970):

0 = No plaque.

1 = Separate areas of plaque at the cervical margin.

2 = A thin continuous band of plaque at the cervical margin (≤1 mm).

3 = Plaque covering <1/3 of the tooth (>1 mm).

4 = Plaque covering >1/3 but <2/3 of the tooth.

5 = Plaque covering 2/3 or more of the tooth.

### 2.7 Statistical analysis

Statistical analysis was performed using IBM SPSS software version 24 (IBM Corp.). Kolmogorov–Smirnov test was used to determine the normality of data. Regarding demographic profile, Fisher's exact test was used for comparing sex, and caries risk and one‐way ANOVA test was used for comparing patient age among study groups. For comparison of the TMQHPI scores between groups at different time points, the Kruskal–Wallis test was applied. Wilcoxon signed‐rank test was performed to compare the dental plaque accumulation between the different time points for each group. Statistical significance was adjusted at 0.05.

## 3 RESULTS

Based on selection criteria, out of 56 patients who were assessed for eligibility, 45 were selected. Approximately more than half of the patients (53.30%) were female, and more than two‐thirds of them (71.10%) were at high risk of caries. The mean age was 29.42 (SD 3.68; range 24–36 months) as listed in Table 1. No significant difference was found between the study participants regarding their demographic characteristics (*p* > .05). In addition, no statistically significant difference was noted between TMQHPI scores at t_0_ (*p* = .789) (Table 2) suggesting that the entire baseline data were homogenous. Regarding t_1_ and t_2_, no statistically significant difference was found in the TMQHPI scores between the three groups (*p* > .05) (Table 2).

**Table 1:** Characteristics of the study participants.

Characteristics	Total (*n* = 45)	DW (*n* = 15)	CHX (*n* = 15)	PVP‐I (*n* = 15)	*p* Value
Sex					.176
Female *n* (%)	24 (53.30)	7 (46.70)	6 (40.00)	11 (73.30)	
Male *n* (%)	21 (46.70)	8 (53.30)	9 (60.00)	4 (26.70)	
Age (months)					.909
Mean ± SD	29.42 ± 3.68	29.40 ± 3.78	29.73 ± 3.67	29.13 ± 3.83	
Min–max	24–36	25–35	24–35	25–36	
Caries risk					.602
Low *n* (%)	3 (6.70)	2 (13.30)	1 (6.70)	0 (0.00)	
Moderate *n* (%)	10 (22.20)	4 (26.70)	2 (13.30)	4 (26.70)	
High *n* (%)	32 (71.10)	9 (60.00)	12 (80.00)	11 (73.30)	

*Note*: Fisher's exact test was used for sex, and caries risk and a one‐way ANOVA test was used for patient age.

Abbreviations: ANOVA, analysis of variance; CHX, chlorhexidine; DW, distilled water; Max, maximum; Min, minimum; n, sample size; PVP‐I, povidone‐iodine; SD, standard deviation.

**Table 2:** Comparison results of the Kruskal–Wallis test of the TMQHPI scores between groups at different time points.

Time points	Groups	*p* Value
t_0_	DW	.789
	CHX	
	PVP‐I	
t_1_	DW	.822
	CHX	
	PVP‐I	
t_2_	DW	.332
	CHX	
	PVP‐I	

Abbreviations: CHX, chlorhexidine; DW, distilled water; PVP‐I, povidone‐iodine; t_0_, baseline; t_1_, at Day 3; t_2_, at Day 7; TMQHPI, Turesky‐modified Quigley‐Hein plaque index.

**Figure 2:**
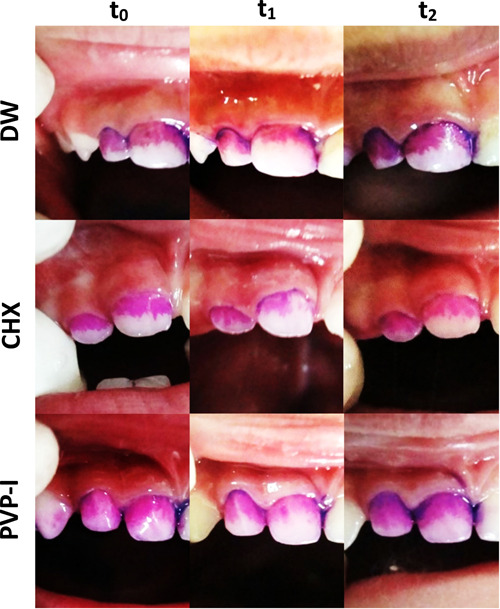
Dental plaque accumulation at different time points among the study groups.

Table 3 shows the descriptive statistics of the TMQHPI scores at different time points of follow‐up between the groups. In the DW group, the mean of the TMQHPI score was 1.89 ± 0.67 at t_0_ and decreased to 1.45 ± 0.66 at t_1_ (*p* = .028). However, the TMQHPI score increased to 1.83 ± 1.06 at t_2_ but this change was not significant (*p* = .078) (Table 4). Similarly, in the CHX group, the mean of the TMQHPI score was 1.83 ± 1.06 at t_0_ and decreased to 1.02 ± 0.99 at t_1_ (*p* = .033), then increased to 1.29 ± 0.74. However, no statistically significant difference was noted in the TMQHPI score at t_2_ (*p* = .438) (Table 4). Regarding the PVP‐I group, the mean of the TMQHPI score went from 1.84 ± 0.85 to 1.01 ± 0.61 at t_1_ then increased to 1.57 ± 0.74 at t_2_. Those changes were statistically significant (*p* = .001) and (*p* = .002), respectively (Table 4) (Figure 2).

**Table 3:** Descriptive statistics of TMQHPI scores at the different time points between the study groups.

Group	*N*	Time point	Mean	SD	SE	Min.	Max.
DW	15	t_0_	1.89	0.67	0.17	0.7	2.8
		t_1_	1.45	0.66	0.17	0	2.3
		t_2_	1.83	1.06	0.27	0	3.1
CHX	15	t_0_	1.83	1.06	0.27	0	3.1
		t_1_	1.02	0.99	0.26	0	3.8
		t_2_	1.29	0.74	0.19	0	2.8
PVP‐I	15	t_0_	1.84	0.85	0.22	0.6	3.4
		t_1_	1.01	0.61	0.16	0.3	2.3
		t_2_	1.57	0.74	0.19	0.5	3.4

Abbreviations: CHX, chlorhexidine; DW, distilled water; Max., maximum; Min., minimum; N, sample size; PVP‐I, povidone‐iodine; SD, standard deviation; SE, standard error; t_0_, baseline; t_1_, at Day 3; t_2_, at Day 7.

**Table 4:** Comparison results of Wilcoxon signed‐rank test dental plaque accumulation between the different time points.

Groups	Time points	Mean difference	*p* Value
DW	t_0_ vs. t_1_	−0.44	0.028*
	t_0_ vs. t_2_	−0.55	0.842
	t_1_ vs. t_2_	0.39	0.078
CHX	t_0_ vs. t_1_	−0.81	0.033*
	t_0_ vs. t_2_	−0.54	0.115
	t_1_ vs. t_2_	0.27	0.438
PVP‐I	t_0_ vs. t_1_	−0.83	0.001*
	t_0_ vs. t_2_	−0.27	0.106
	t_1_ vs. t_2_	0.56	0.002*

Abbreviations: CHX, chlorhexidine; DW, distilled water; n, sample size; PVP‐I, povidone‐iodine; t_0_, baseline; t_1_, at Day 3; t_2_, at Day 7.

* *p* < .05 = significant difference.

## 4 DISCUSSION

CHX gel and PVP‐I solution are two commonly used antiplaque agents, but their efficacy in toddlers has not been extensively studied. The current randomized controlled trial was conducted to compare the efficacy of CHX gel and PVP‐I solution in reducing plaque regrowth in toddlers aged 24–36 months.

Mechanical plaque control is the cornerstone for preventing gingivitis and caries. However, it requires patient compliance and motivation making it less effective in controlling dental plaque. Therefore, chemical plaque control using antimicrobial agents seems promising. However, the long‐term use of some antiplaque agents can lead to bacterial resistance so clinicians should pay close attention when applying antimicrobial agents several times (Anil et al., 2016). This explains the one‐time use of chemical antiplaque agents adjacent to the mechanical effect of the cotton‐tipped swab in the current study. Antiplaque agents should mainly be applied by a pediatric dentist in a knee‐to‐knee position due to the lack of compliance on the part of toddlers (Fux et al., 2019).

In this study, CHX was used as an antiplaque agent due to antimicrobial efficacy, substantivity, and safety. In addition, it is the gold standard in terms of chemical plaque control (Balagopal & Arjunkumar, 2013; Chapman et al., 2012). CHX gel form was used because it is effective and provides a long‐lasting release. It is also safe and easy to use, making it a preferred option for many dentists (Ahmedi et al., 2023). PVP‐I is a common antiseptic used in dentistry that can be found in many over‐the‐counter products. It is a broad‐spectrum disinfectant that is effective against both Gram‐positive and Gram‐negative bacteria (Eggers, 2019; Lepelletier et al., 2020). PVP‐I works by breaking down plaque and removing bacteria. However, the prolonged use of PVP‐I seems unfavorable due to its absorption through the oral mucosa (Anil et al., 2016). In the current study, PVP‐I as an antiplaque agent was not superior to CHX (positive control) nor to DW (negative control) in terms of plaque regrowth inhibition. This result could be explained by the fact that the two antiplaque agents are similar in their antimicrobial efficacy (Eggers, 2019; Lepelletier et al., 2020). In addition, according to Narayan et al. (2017), CHX varnish was not superior to 10% PVP‐I in suppression of plaque and saliva Streptococcus mutans. According to the study by Twetman and Grindefjord (1999), 1% CHX gel was used twice daily in toddlers aged 12–18 months, and CHX suppressed the levels of Streptococcus mutans. In addition, Wan et al. (2003) concluded that applying 0.2% CHX gel once a week for 3 months in toddlers aged 10 months was also effective in reducing Streptococcus mutans. However, according to Neeraja et al. (2008), CHX was more effective than PVP‐I when used as mouth rinses for 3 months. Regarding PVP‐I efficacy, Berkowitz et al. (2009) found that using 10% PVP‐I in children aged 2–5 years was effective in suppressing Streptococcus mutans for 90 days. However, in the current study, the antiplaque agents were applied one time suggesting the need for further trials to evaluate the efficacy of their repeated applications.

In the CHX group, there was a statistically significant decrease in the TMQHPI mean scores at t_1_. However, the TMQHPI mean scores increased at t_2_. This result could be explained by the fact that CHX has a long‐lasting effect only when it is applied intensively; 3–4 times per day or 10 to 14 daily applications (Ribeiro et al., 2007). Thus, the current result is in agreement with the findings of Slot et al. (2014), which suggested that brushing with CHX gel was not effective in dental plaque control. In addition, Plonka et al. (2013) found that brushing with 0.12% CHX gel was not superior to 10% casein phosphopeptide‐amorphous calcium phosphate paste in terms of controlling ECC. On the contrary, Slot et al. (2007) found 1% CHX gel applied with trays was superior to 0.12% CHX dentifrice gel and to 0.2% CHX mouthwash. This variation could be attributed to the fact that using trays ensures that CHX gel can reach all tooth surfaces simultaneously when compared with a cotton‐tipped swab.

In the PVP‐I group, there was a statistically significant decrease in the TMQHPI mean scores at t_1_, then an increase at t_2_. However, PVP‐I was not superior to DW in plaque control. This result could be attributed to the difficulty in achieving optimal moisture control. According to Reilly et al. (2016), the repeated use of a 10% PVP‐I and 5% sodium fluoride varnish combination led to optimal plaque control. Furthermore, according to several studies, combining PVP‐I and sodium fluoride varnish lead to Streptococcus mutans suppression (Hashemi et al., 2015; Milgrom et al., 2021). According to Simratvir et al. (2010), the repeated use of 10% PVP‐I yielded satisfactory outcomes in terms of preventing dental caries and reducing Streptococcus mutans counts. In addition, according to Lopez et al. (2002), the topical application of 10% PVP‐I improves disease‐free survival in toddlers with high caries risk. Furthermore, Amin et al. (2004) suggested that PVP‐I was effective in reducing Streptococcus mutans counts at 6 months. Therefore, the repeated application of PVP‐I is beneficial.

This study has limitations. First, the short follow‐up period of the current study. Second, the single application of the antiplaque agents. Therefore, there is a need for further studies to evaluate the efficacy of their repeated use.

## 5 CONCLUSIONS

CHX and PVP‐I efficacy lasted for 3 days, and PVP‐I did not demonstrate superior plaque control to CHX in toddlers. Chemical plaque control agents should be considered an adjunct to mechanical methods, not an alternative. Further studies are needed to confirm these findings and to determine the long‐term efficacy and safety of these antiplaque agents in toddlers.

## AUTHOR CONTRIBUTIONS

Nour Al Basha collection and/or assembly of data, data analysis and interpretation. Mawia Karkoutly writing the article. Nada Bshara research concept and design, critical revision of the article, and final approval of the article.

## CONFLICT OF INTEREST STATEMENT

The authors declare no conflict of interest.

## ETHICS STATEMENT

Ethical approval was provided by the ethics board at Damascus University.

## Data Availability

The data sets generated during and/or analyzed during the current study are available from the corresponding author upon reasonable request.
